# Physiological and Biochemical Responses of *Medicago sativa* L. Infected by *Cuscuta* sp.

**DOI:** 10.3390/life15121892

**Published:** 2025-12-11

**Authors:** Hümeyra Ayvacı, Mehmet Ertuğrul Güldür, Murat Dikilitas

**Affiliations:** Department of Plant Protection, Faculty of Agriculture, Harran University, Şanlıurfa 63300, Turkey

**Keywords:** *Cuscuta* sp., *Medicago sativa*, parasitic weed, holoparasite, host–parasite interaction

## Abstract

This study aims to elucidate the physiological and biochemical alterations induced by parasitic *Cuscuta* sp. (dodder) in lucerne (*Medicago sativa* L.), a key forage crop. Comparative analyses between infected and healthy plants revealed that significant reductions in chlorophyll *a*, *b*, and total chlorophyll, and protein levels in the leaf and stem tissues of *Cuscuta*-infested plants were evident. The parasitic infection led to increased activities in antioxidant enzymes such as catalase (CAT) and peroxidase (POX) in stems, but not in leaves. Phenolic compounds were significantly lower both in leaves and stems of dodder-infected lucerne plants. No statistically significant changes were detected in jasmonic acid (JA) and salicylic acid (SA) levels in both plant parts, suggesting that classical defense signaling pathways may not be predominantly activated under *Cuscuta*-mediated stress. Possibly, host defense might be impaired. Histological examinations demonstrated active structural defense responses, including localized tissue remodeling and the formation of callose-like structures at haustorial penetration sites. DNA fragmentations showed that *Cuscuta*-infected *M. sativa* plants exhibited slightly higher instability. Collectively, these findings provide novel insights into the molecular and biochemical basis of the *Cuscuta*-lucerne interactions and highlight the need for further investigation into host defense mechanisms. We assume that active defense structural parts at early growth stages of lucerne or hypersensitive-type responses occurring in the early penetration phase might fend off the invading holoparasite. The results also offer a valuable foundation for the development of *Cuscuta*-resistant lucerne cultivars and support the design of integrated, sustainable weed management strategies to mitigate the detrimental effects of parasitic plants on forage production systems.

## 1. Introduction

Lucerne (*Medicago sativa* L.), a perennial leguminous forage crop of the Fabaceae family, is recognized as one of the most widely cultivated and nutritionally valuable plant species in global agriculture. Its high dry matter yield, rich protein content, and superior digestibility make it a cornerstone in ruminant nutrition. Beyond its forage quality, lucerne contributes significantly to agroecosystem sustainability through its extensive root system, which enhances soil structure by improving aeration, reducing compaction, and limiting erosion. Additionally, it can fix atmospheric nitrogen via symbiosis with *Rhizobium* spp., and it enriches soil fertility and reduces the need for synthetic fertilizers [1]. Despite these agronomic and ecological advantages, lucerne cultivation is highly susceptible to a range of abiotic (e.g., drought, salinity, temperature extremes) and biotic (e.g., pathogens, pests, parasitic plants) stress factors. Among biotic threats, species of *Cuscuta* sp. (dodder) have recently gained attention due to their aggressive parasitic nature and destructive impact on plant health and productivity [2,3]. “Dodders” is the common name for a group of holoparasitic plants of the angiosperm genus *Cuscuta* spp. Since every infection starts with the parasite coiling around the host stem and gluing itself onto its host surface with a sticky mucilage, the infection becomes more severe when compared to those of any fungal pathogens due to the single infection point being the main route to pathogenic infection. Therefore, suppression of host defense created by holoparasite plants could be much easier when compared to those of other infection strategies. Here, the haustoria break through the host surface and grow until they have reached the vascular tissue, and spread laterally and apically via finger-like scavenging cells called “feeding hyphae”. During penetration, the haustoria soften and decompose the middle lamellae of the host to suppress defense response. For a successful infection, *Cuscuta* has to inhibit the signaling mechanisms of the host. Dodders can also form phloem and/or xylem fusion in the host vascular systems. In this way, dodders can take up minerals, water, and organic compounds at the same time from the host. It could nourish itself with organic compounds while decreasing the defense mechanisms of the host via transferring the secondary metabolites, messenger RNAs, micro RNAs, amino acids, proteins, and noncoding RNAs to suppress the signaling molecules in the host [4].

Members of the dodder genus, belonging to the Convolvulaceae family, are obligatory holoparasitic plants devoid of chlorophyll and photosynthetic pigments. They are entirely reliant on host plants for water, nutrients, and organic metabolites, and they absorb materials through specialized invasive structures known as haustoria [5]. The economic loss caused by holophyte parasitic weeds on crop plants could be expressed in hundreds of millions of US Dollars around the globe, and this figure is enormously increasing every year [6].

The establishment of haustorial connections by *Cuscuta* sp. leads to severe physiological and biochemical disruptions in the host plant [7,8]. These include the depletion of vital resources, suppression of photosynthesis, and ultimately, significant reductions in biomass [9,10]. Parasitic plants coil around the stems and branches of the host plant and use a special organ called a haustorium to attach to the host plant and extract water and nutrients from it [11]. The haustoria, specialized invasive structures, penetrate the host’s epidermal and vascular tissues to connect with the vascular system [12,13]. *Cuscuta* spp. are rootless and leafless holoparasitic plants on crop plants. They have vine-like stems that twine around the host stem and selectively take nutrients and genes from the host [12]. Infection strategies of parasitic plants are quite similar to microbial plant pathogens [14,15]. Therefore, they must interact closely with host plants to extract nutrients and suppress defense responses. Although plants possess large arsenals of immune receptors capable of recognizing all pathogen classes, invading pathogens, including holoparasites, suppress or evade immune perception and drive nutrients from host tissues [16].

Dodder infection markedly lowers chlorophyll *a* and *b* levels, reduces total protein content, and alters antioxidant defense pathways by increasing the activity of key enzymes such as catalase (CAT) and peroxidase (POX) as well as inducing phenolic compound biosynthesis [17,18]. These responses are believed to reflect the defense capacity of host plants against the oxidative stress triggered by parasitic invasion [19,20,21]. In addition to biochemical responses, structural defense mechanisms have also been documented, including localized tissue remodeling and the formation of callose-like structures at infection sites—suggesting a targeted response to haustorial penetration [22].

Dodder parasitism has also been shown to trigger a multilayered oxidative stress response in the host plant. All compounds, hormones, and enzymes are present in plants, both healthy and infected. Infection or other stress conditions usually lead to changes in enzyme activity or compound levels [23]. Under normal conditions, ROS generation is usually compensated for by the action of antioxidant enzymes or low-molecular-weight antioxidants and therefore does not lead to damage to cellular structures [24]. Plants could only cope with this stress upon activating both enzymatic and non-enzymatic antioxidant defense systems. Dodder parasitism could be intensified under other biotic or abiotic stress conditions. For example, Shuo Duan et al. [25] reported that salt stress increased the dodder infection and significantly modulated secondary metabolism and stress-responsive genes in *Citrus sinensis* plants. Fareed et al. [26] reported that superoxide dismutase (SOD) and catalase (CAT) enzymes increased at the infection point of tomato plants, followed by *Cuscuta chinensis* invasion. The authors suggested that *Cuscuta*-induced oxidative stress in tomato plants led to increased SOD and CAT activity, which counteracted the damage resulting from ROS. They also showed that the MDA level of tomato plants significantly increased. Increases in antioxidant enzymes were more prevalent and higher in the parasitized sites than in other parts of the plant.

Van Wuller et al. [27] stated that hormones acted as signaling molecules and were systematically transported between cells, organs, tissues, and between parasite and host. The interaction between *Cuscuta* species and their host plants also triggers a complex biotic stress response that leads to significant alterations in hormone levels and defense-related gene expression.

Plant hormones are signaling molecules regulating crucial mechanisms involved in growth and development in biotic and abiotic stresses [28]. Regulation of plant defense mechanisms mainly involves the phytohormones jasmonic acid (JA) and salicylic acid (SA). In general, JA-mediated defense response is involved against herbivores, while the SA-mediated pathway is involved in stress caused by microbial pathogens [29]. However, other hormones are also involved, such as abscisic acid (ABA) and cytokinins. For example, Furuhashi et al. [30] reported that *Cuscuta* parasitism resulted in changes in cytokinin levels in alfalfa. Spallek et al. [31] reported that cytokinin levels significantly increased in *Arabidopsis thaliana* following the inoculation of holoparasite plant *Phtheirospermum japonicum*. They revealed that transport of parasite-derived cytokinins resulted in physiological and morphological modifications in host roots. Furuhashi et al. [32] later stated that not only are hormones involved in haustorial penetration, but blue light is also essential for twinning around the plants. They suggested that blue light and auxin hormone combinations could ease the penetration of *Cuscuta* spp. Other hormones also have great parts in *Cuscuta* spp. invasions. For example, a specific signaling pathway involved in *Cuscuta campestris* invasion was partly revealed by Narukawa et al. [33], who stated that ethylene synthesis in host plants altered infection phenotype.

Regarding the JA and SA pathway, we know that these hormones are involved in hautoria attachment and disease development. For example, Bar-Nun and Mayer [34] stated that methyl jasmonate or methyl salicylate increased host resistance to *Orobanche aegyptiaca*. Recently, Yang et al. [6] reported that *Cuscuta pentagona* and *C. australis* parasitism induced JA and SA accumulation in their tomato hosts, which activated the hypersensitive response. However, van Wullen et al. [27] reported that no increase in either JA or SA was observed during hemiparasite plant *Phtheirospermum japonicum* infection. They revealed that JA and SA hormones might have a significant role in host response to parasitic plants but a minor role in haustorium development. They stated that parasitic plants might have developed a mechanism to avoid JA and SA accumulation during haustoria development to avoid inducing the host defense responses triggered by haustorial invasion.

Interactions between parasitic plants and their hosts have been revealed in molecular detail. Zhou et al. [28] reported that they identified 1329 and 3271 differentially expressed genes (DEGs) in the leaf and root tissues of white clover (*Trifolium repens* L.), and they revealed the pathways through plant hormone signal transduction and phenylpropanoid biosynthesis pathways. WRKY, AP2/ERF, bHLH, bZIP, MYB, and NAC transcription factors showed a close relationship with lignin synthesis-related genes. Yang et al. [6] reported that horizontal gene transfer (HGT) was observed between *Cuscuta* and its hosts, as observed between fungal pathogens and the host plants. It is known that the transfer of genetic material between pathogens and their hosts forms the basis of resistance. The authors stated that 108 genes transferred to the dodder’s genome were involved in parasitism, haustorial structure, and amino acid metabolism, and microRNAs (miRNAs) produced in the cell metabolism of dodders were sent back to the host plant to suppress defense through gene silencing. Although major pathways have been revealed, followed by dodder infection, how dodders find host plants and locate the infection points and carry out pathogenicity without significant defense responses remains elusive. Recent studies showed that the mechanism of defense suppression is more complex than previously thought. For example, Shahid et al. [35] showed that *Cuscuta* campestris accumulated high levels of many novel miRNAs while parasitizing *Arabidopsis thaliana* through haustoria. Many of these miRNAs decreased mRNA accumulation in *A. thaliana*. The authors suggested that these miRNAs acted as virulence factors during parasitism.

The cumulative effects of such infections lead to stunted growth, reduced forage quality, and diminished yield potential, posing a significant threat to the economic viability of lucerne-based forage systems [36,37]. If *Cuscuta* sp. damage reaches the DNA molecule, the recovery of host plants might be extremely difficult since all metabolic activities are coded there. Therefore, any damage and instability in DNA molecules affects the whole metabolism.

Given the increasing prevalence of dodder infestations and their substantial agronomic impact, there is a critical need to better understand the dodder–lucerne interactions at molecular, physiological, and biochemical levels. In this study, comparative analyses of healthy and infected lucerne plants are conducted to evaluate the physiological and biochemical consequences of parasitic stress. Insights gained from such studies are essential for the development of resistant lucerne cultivars and play a vital role in designing innovative and sustainable crop management strategies in the face of parasitic weed challenges.

## 2. Materials and Methods

### 2.1. Plant Materials and Sampling Procedure

The study area contained clay, sand, and silt, ranging from 37.5 to 54.17%, from 20.8 to 27%, and from 20.83 to 33.33%, respectively. The soils were slightly alkaline, with pH values between 7.51 and 8.16. Organic matter content was low (0.61–2.37%), whereas calcium carbonate content was high (46.01–54.7%). Electrical conductivity (EC) ranged from 0.14 to 0.80 dS/cm, indicating no salinity problem. Available phosphorus content varied between 2.52 and 5.86 kg/da [38]. The average monthly temperatures in the experimental area covering the plantation of lucerne ranged between 7.1 and 32 °C, between February and June. The average monthly precipitation was 66.3 mm in February, 57.9 mm in March, 44.7 mm in April, 26.2 mm in May, 5.8 mm in June, and 2.0 mm in July.

Lucerne (*Medicago sativa* L. cv. Alsancak) seeds were sown in an experimental field plot (3 m × 3 m) in Harran University, Şanlıurfa, Türkiye. *Cuscuta* sp. seeds were artificially inoculated within the roots of lucerne by mixing with soil. Within weeks, stem colonization of lucerne by dodder was observed. For the analyses, mature intermediate leaves and stem samples were assessed separately in all biochemical analyses. The stem samples were collected from the regions where dodder formed the highest density of haustoria. The experimental trial was carried out in 2023. The lucerne plant samples were first collected at the 90th day, followed by 15-day intervals, for the growth parameters from the dodder-infested and the control plots. All samples were carefully labeled in detail, including sampling location, date, and observed symptoms, and the leaf and stem samples were stored at −20 °C until analysis. Height, fresh and dry weights of the plants were recorded at each sampling time. For the biochemical analysis, the samples were collected at the final harvest stage.

### 2.2. Yield and Growth Measurements

Height measurements (cm) of lucerne plants were made on dodder-infested and non-infested plots by measuring the height from the soil surface to the plant apex. To determine green forage yield, plants were harvested from 10 different locations, and their fresh weights (Fwt) were recorded. The same samples were then oven-dried at 70 °C for 72 h, and dry forage yield (Dwt) was determined thereafter. Yield values from infested and non-infested plots were recorded and converted to yield da^−1^.

### 2.3. Biochemical Analysis

Biochemical analyses were performed on *Cuscuta* sp.-infected and non-infected plants. To minimize standard errors arising from biological variability among samples, a single representative plant was selected from each experimental group. This approach not only enhanced the accuracy of the biochemical data obtained from both infected and healthy groups but also ensured their comparability.

#### 2.3.1. Determination of Protein Content

To quantify protein content, approximately 0.5 g of fresh leaf tissue was homogenized in 5 mL of 50 mmol L^−1^ sodium phosphate buffer (pH 7.0). From the resulting plant extract, 100 μL was mixed with 5 mL of Coomassie Brilliant Blue G-250 dye reagent. The absorbance of the mixture was measured spectrophotometrically at 595 nm. Protein concentration was quantified by constructing a standard curve using Bovine Serum Albumin Fraction V (Sigma, St. Louis, MO, USA) with concentrations ranging from 10 to 100 μg mL^−1^.

#### 2.3.2. Determination of Chlorophyll Content

Chlorophyll contents in lucerne leaves were determined following the method of Arnon [39] with minor modifications as described by Karakaş et al. [40]. Approximately 0.5 g of fresh leaf tissue was homogenized in 5 mL of 80% acetone solution (acetone–water, 80:20 *v*/*v*). The resulting suspension was filtered through filter paper to separate the liquid phase, which was then transferred into light-proof tubes. Chlorophyll *a* and *b* concentrations were measured spectrophotometrically at wavelengths of 663.5 nm and 645 nm, respectively, against a blank solution prepared with 80% acetone (Epoch-BioTek spectrophotometer, BioTek Instruments, Winooski, VT, USA). Total chlorophyll content was calculated in mg fresh weight leaf tissue with the following equations.

(1) Total Chlorophyll (mg L^−1^) = 20.2 A_625_ + 8.02 A_663.5_ Chlorophyll *a* (mg L^−1^) = 12.7 A_663.5_ − 2.69 A_645_ Chlorophyll *b* (mg L^−1^) = 22.9 A_645_ − 4.68 A_663.5_

#### 2.3.3. Determination of Total Phenolic Content

Total phenolic content was measured using a modified version of the method described by Shetty et al. [41]. Approximately 0.5 g of lucerne leaf tissue was homogenized in 80% methanol and incubated at 95 °C for 30 min, followed by centrifugation at 10,000× *g* for 10 min. For the phenolic assay, 300 μL of the extract was mixed with 1.5 mL of diluted (1:10) Folin–Ciocalteu reagent (Merck KGaA, Darmstadt, Germany). After 5 min, 1.2 mL of 20% sodium carbonate (Na_2_CO_3_) was added, and the mixture was incubated in the dark at 40 °C for 30 min. The absorbance of the resulting solution was measured spectrophotometrically at 760 nm, and total phenolic content was calculated using a gallic acid standard calibration curve.

#### 2.3.4. Peroxidase (POX, E.C. 1.11.1.7) Activity Assay

Peroxidase (POX) activity was determined using the method of Cvıkorová et al. [42] with minor modifications [43]. Approximately 0.5 g of leaf tissue was homogenized in 50 mmol L^−1^ phosphate buffer (pH 7.0). Subsequently, 100 μL of the extract was added to a 3 mL reaction mixture containing 13 mmol L^−1^ guaiacol, 5 mmol L^−1^ H_2_O_2_, and 50 mmol L^−1^ sodium phosphate buffer (pH 6.5). The enzymatic reaction was initiated by the addition of H_2_O_2_, and the increase in absorbance was recorded spectrophotometrically at 470 nm every minute for three consecutive measurements at 25 °C. POX activity was expressed as the change in absorbance (ΔA_470_) of 0.1 per minute and reported as units per mg of protein.

#### 2.3.5. Catalase (CAT, E.C. 1.11.1.6) Activity Assay

Catalase (CAT) activity was measured following the method described by Milosevic and Slusarenko [44] with minor modifications introduced by Karakaş [45]. Approximately 0.5 g of leaf tissue was homogenized in phosphate buffer at pH 7.0. From the resulting supernatant, 50 μL was mixed with a 2.95 mL reaction mixture containing 10 mmol L^−1^ H_2_O_2_, 50 mmol L^−1^ phosphate buffer (pH 7.0), and 4 mmol L^−1^ Na_2_EDTA. The decrease in absorbance was recorded spectrophotometrically at 240 nm for 30 s at 25 °C. Enzyme activity was defined as the amount of enzyme decomposing 1 μmol of H_2_O_2_ per minute [4] and expressed as units per mg of protein.

#### 2.3.6. Determination of Jasmonic Acid (JA) Content

Determination of jasmonic acid (JA) content was carried out according to the method described by Annigeri et al. [46]. Initially, 1 g of fresh leaf tissue was extracted with 10 mL of absolute ethanol and incubated in the dark at room temperature for 12 h. At the end of the extraction period, the mixture was filtered through Whatman No. 1 filter paper, and 1 mL of the filtrate was used to measure absorbance at 323 nm using a spectrophotometer (Epoch-BioTek). The JA concentration in the samples was calculated based on a standard curve prepared from known concentrations of JA dissolved in absolute ethanol.

#### 2.3.7. Determination of Salicylic Acid (SA) Content

Salicylic acid (SA) content was determined with slight modifications based on the method of Rainsford [47]. One gram of leaf sample was incubated in 10 mL of ethanol in the dark at room temperature for 12 h, then centrifuged at 10,000× *g* for 10 min. A 100 μL aliquot of the supernatant was mixed with 1% ferric chloride solution, and the volume was adjusted to 3 mL. The violet Fe^3+^-SA complex formed was measured spectrophotometrically at 540 nm. SA concentrations were calculated using a standard curve prepared within the range of 0–100 ppm. The reliability of the method is supported by the high solubility of SA and its ability to react readily with various solvents.

### 2.4. Histological Analysis of Cuscuta sp. Infection on Medicago sativa L.

Histological analysis was made in lucerne plants from the infection point of *Cuscuta* sp., followed by 20 days of infection. The cut-stem section around the infection point was fixed using formalin–acetic acid–ethanol (1:1:1 volume) solution. To enhance visualization of cellular structures, the prepared sections were stained with Safranin-O/Fast Green (Merck KGaA, Darmstadt, Germany) or Toluidine Blue (St. Louis, MO, USA). For this, samples were initially fixed in formalin–acetic acid–ethanol solution and subsequently dehydrated through a graded ethanol series. The tissues were then embedded in paraffin, and 7–10 µm-thick sections were obtained. The sections were stained with Safranin-O/Fast Green or Toluidine Blue, examined under a light microscope, and images were captured using a digital camera. When necessary, histochemical stains such as Periodic Acid-Schiff (PAS) for polysaccharide accumulation and Aniline Blue for detecting callose deposition were applied [48].

The stained sections were examined under a light microscope, and the images were captured using a digital camera [48,49].

### 2.5. Determination of DNA Fragmentation in Medicago sativa and Cuscuta sp.

The pathogenic effect of *Cuscuta* sp. on *Medicago sativa* L. plants was evaluated to find out if DNA integrity was disturbed. DNA fragments were also evaluated in non-infected lucerne and *Cuscuta* sp. plants. The degree of DNA damage or disruption of DNA integrity can provide clues as to whether the plant can compensate for the loss caused by stress, i.e., it can firmly say whether the stressed plant can recover or not. For this purpose, plant genomic DNA was isolated using methods developed by Ahrens and Seemuller [50] and Surapu et al. [51] with minor modifications. Fresh wheat leaves (1 g) were ground in a mortar with 4 mL CTAB buffer (2% *w*/*v* cetyltrimethylammonium bromide, 1.4 mol L^−1^ NaCl, 0.2% β-mercaptoethanol, 20 mmol L^−1^ EDTA, 100 mmol L^−1^ Tris-HCl, 2% polyvinylpyrrolidone, pH 8.0). Followed by the protocol, the resulting DNA solution was dissolved in 80–100 μL Tris-EDTA (TE) buffer (10 mmol L^−1^ Tris-HCl, 1 mmol L^−1^ EDTA, pH 8). The DNA fragments were separated by electrophoresis in a 1% agarose gel (Sigma, Aldrich) prepared in 1×Tris Acetate EDTA (TAE) at 85 V for 75 min, which proved that the yellow color in the loading buffer solution reached the opposite end of the gel tank [52]. (Sigma, Aldrich) prepared in 1×Tris Acetate EDTA (TAE) at 85 V for 75 min. Post-electrophoresis DNA images were obtained by staining with ethidium bromide (1 μg mL^−1^). Since DNA is a molecule visible under UV fluorescent light, the fragmented DNA bands became visible and exhibited ladder-like patterns using the imaging system.

### 2.6. Statistical Analysis

Biochemical data obtained from dodder-infected and non-infected plants were analyzed using Minitab 20.1 software (Minitab Inc., State College, PA, USA) [53]. Results are presented as mean ± standard error (*n* = 3). Statistical comparisons and relevant tests were performed to assess significant differences (*p* < 0.05) between treatment groups.

## 3. Results

### 3.1. Yield and Growth Measurements

**Figure 1:**
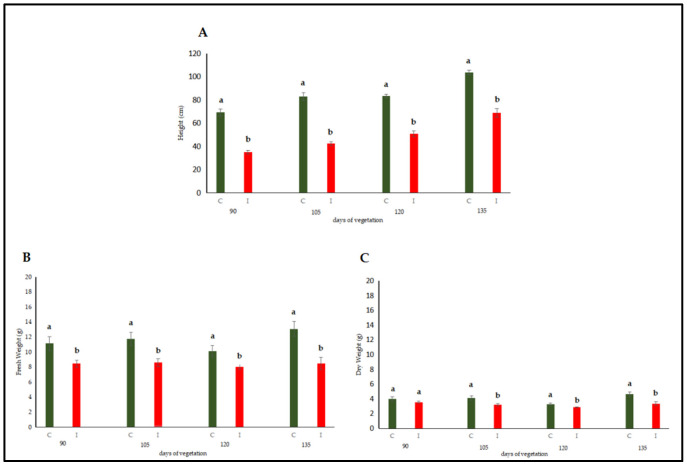
Evaluation of stem length measurements (**A**), fresh weight (**B**), and dry weight (**C**) of *Cuscuta* sp.-infected lucerne (*Medicago sativa* L. cv. Alsancak) plants at the 90th, 105th, 120th, and 135th day post-sowing. Different letters above the bars indicate statistically significant differences from the control plants based on the Least Significant Difference (LSD) test at *p* < 0.05 (*n* = 3).

The initial plant height measurement was conducted on the 90th day after sowing; the subsequent measurements were made at 15-day intervals on the 105th, 120th, and 135th day. Based on the data, the shoot lengths of lucerne (*Medicago sativa* L. cv. Alsancak) plants infected with *Cuscuta* sp. were found to be significantly lower when compared to non-infected plants (Figure 1A). The height of *Cuscuta*-infected plants was almost 50% lower at the 90th day of the experiment, and this range was maintained towards the end of the experimental trial, although the height of infected plants was a bit taller at the end of the trial.

The harvest commenced at the 90th day post-sowing, with a total of four harvests conducted at 15-day intervals (on the 105th day, 120th day, and 135th day). Analysis of data revealed that both fresh and dry forage yields of dodder-infested lucerne plants were significantly lower than those of non-infected plants (Figure 1B,C). The infected plants were only 80% of the control plants, and this ratio was relatively lower at the end of the experiment, indicating that lucerne plants could not accumulate proteins and carbohydrates that could contribute to the increase in fresh weight.

The results regarding fresh and dry weight were converted to kg yield per da area. When conversion was made, the average green herbage yield in the dodder-infected area was measured at 847.7 kg/da, while the dry herbage yield was 324.64 kg/da. In contrast, the corresponding values for the uninfected control plants were recorded as 1153.43 kg/da and 402.22 kg/da, respectively.

### 3.2. Biochemical Analysis

#### 3.2.1. Chlorophyll and Protein Contents

Chlorophyll and protein contents of leaves and stems of dodder-infected plants were assessed. According to the results, total chlorophyll and chlorophyll *a* and *b* contents in infected lucerne leaves and stems were significantly lower when compared to those of healthy plants. The significant decrease in chlorophyll *b* also contributed to the decrease in total chlorophyll content. When comparison was made, the chlorophyll contents of leaves were preserved when compared to those of stems, indicating that the invasion of Cuscuta through stems targeted chlorophyll contents in stems, and Cuscuta possibly used chlorophyll proteins and other carbohydrates as the first energy source.

**Figure 2:**
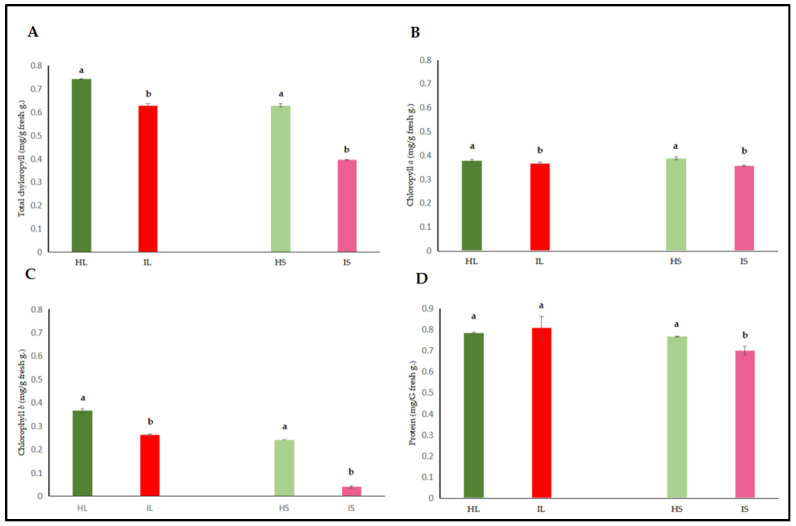
Total Chlorophyll (**A**), chlorophyll *a* (**B**), chlorophyll *b* (**C**), and protein (**D**) contents were evaluated in the leaf and stem tissues of lucerne (*Medicago sativa* L. cv. Alsancak) infected with *Cuscuta* sp. Different letters above the bars indicate statistically significant differences according to the Least Significant Difference (LSD) test at *p* < 0.05 (*n* = 3).

Although protein contents were low in infected plants, no statistical differences were observed between the infected and healthy leaves (Figure 2A–C). In contrast, a statistically significant decrease in protein content was detected when infected and healthy stems were compared (Figure 2D).

#### 3.2.2. Quantitative Evaluation of Phenolic Compounds

**Figure 3:**
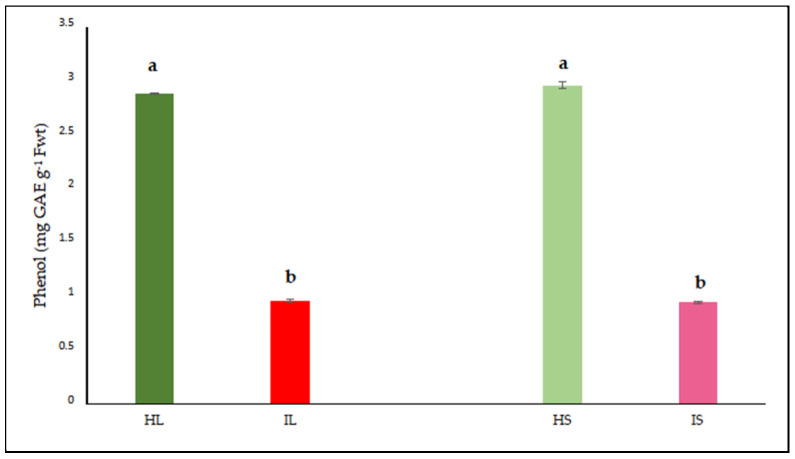
Phenol was evaluated in the leaf and stem tissues of lucerne (*Medicago sativa* L. cv. Alsancak) infected with *Cuscuta* sp. Different letters above the bars indicate statistically significant differences according to the Least Significant Difference (LSD) test at *p* < 0.05 (*n* = 3).

In order to determine the effects of dodder infection on the host plant, the analyses revealed that the phenol content in both tissues of infected lucerne plants was significantly lower when compared to that of healthy plants (Figure 3). Phenolic compounds were not excessively produced, indicating that defense responses towards the end of the experimental trial were minimized both in leaves and stems.

#### 3.2.3. Quantitative Assessment of Antioxidant Enzyme Activities and Hormones

**Figure 4:**
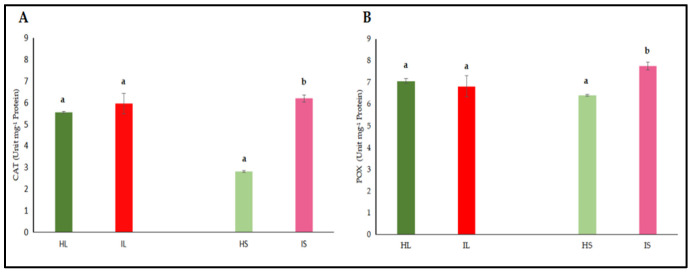
Catalyze (CAT) (**A**) and peroxidase (POX) (**B**) activity were evaluated in the leaf and stem tissues of lucerne (*Medicago sativa* L. cv. Alsancak) infected with *Cuscuta* sp. Different letters above the bars indicate statistically significant differences according to the Least Significant Difference (LSD) test at *p* < 0.05 (*n* = 3).

**Figure 5:**
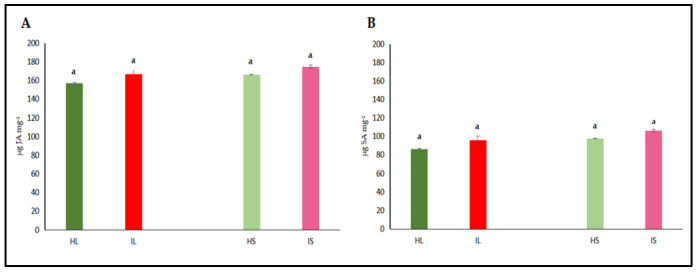
Jasmonic acid (JA) (**A**) and salicylic acid (SA) (**B**) activity were evaluated in the leaf and stem tissues of lucerne (*Medicago sativa* L. cv. Alsancak) infected with *Cuscuta* sp. Different letters above the bars indicate statistically significant differences according to the Least Significant Difference (LSD) test at *p* < 0.05 (*n* = 3).

The activities of catalase (CAT) and peroxidase (POX) enzymes, jasmonic acid (JA), and salicylic acid (SA) were independently assessed in the leaf and stem tissues of dodder-infected and healthy plants. The analyses revealed that CAT and POX activities in the leaf tissues of infected plants did not differ significantly from those of healthy controls (Figure 4A,B). However, CAT and POX activities in the stem tissues of infected plants were significantly higher when compared to healthy controls. On the other hand, it was determined that the levels of JA and SA in both leaf and stem tissues did not show a statistically significant difference from those of control plants, both in leaves and stems (Figure 5A,B). Similar findings to those of phenol accumulation in leaves and stems were also observed with those of findings obtained from antioxidant enzymes. In stems, antioxidant enzymes were higher, indicating that as haustorial penetration carried on, defense responses were also active; however, defense responses were not different from those of healthy controls, indicating that defense metabolites of leaves were not high enough to suppress the pathogenic invasions. Defense-related hormone (JA and SA) accumulation was not significantly high, indicating that the hormonal triggering defense pathway was not significantly activated. When haustorial invasions started, this invasion lasted for almost three months, and defense mechanisms were not at a high level to suppress the pathogenic attack or activate the defense responses of lucerne plants.

#### 3.2.4. Evaluation of Callose Responses Induced by Parasitic Infection in Plants

**Figure 6:**
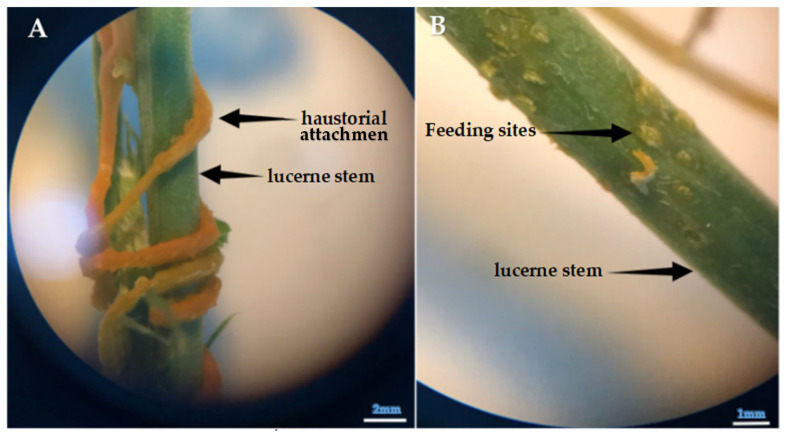
(**A**) Attachment of *Cuscuta* sp. to the stem of lucerne (*Medicago sativa* L. cv. Alsancak) via haustoria attachment. (**B**) Feeding (haustorial) sites that appear on the lucerne stem following the removal of the haustoria (×400 magnification).

**Figure 7:**
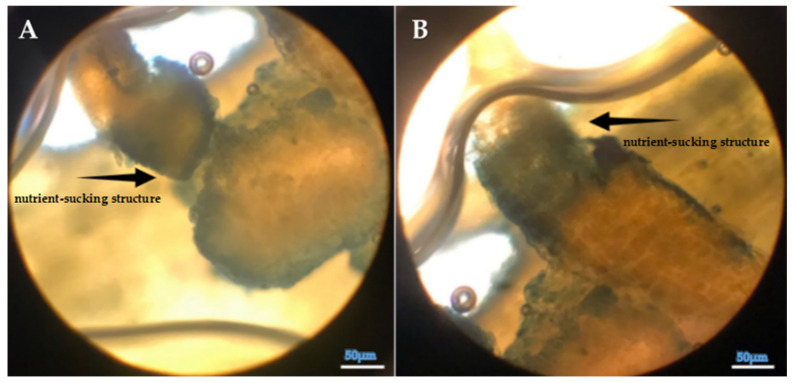
(**A**,**B**) Structural appearance and internal positioning of *Cuscuta* sp. haustoria penetrating the lucerne (*Medicago sativa* L. cv. Alsancak) stem (×400 magnification).

**Figure 8:**
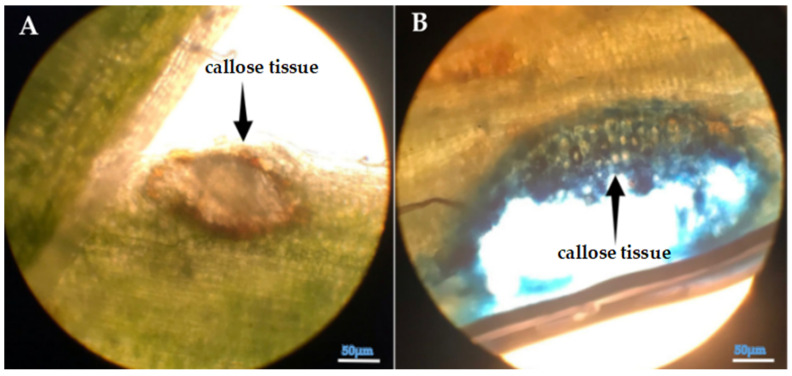
(**A**,**B**) Light microscopy image of the callose tissue developed in the host tissue following the penetration of *Cuscuta* sp. into the (*Medicago sativa* L. cv. Alsancak) stem (×400 magnification).

Microscopic analysis clearly revealed prominent callose formation at the sites where *Cuscuta* sp. penetrated the host plant (Figure 6, Figure 7 and Figure 8). Upon haustorial penetration, we only observed the callose formation around the infection sites, not in the other sites of the stems of lucerne. We assume that systemic defense responses were not activated, as observed in the other pathogenic infections. The results of histological findings were in accordance with the findings of biochemical findings.

#### 3.2.5. Evaluation of DNA Damage in Medicago Sativa Infected with *Cuscuta* sp.

**Figure 9:**
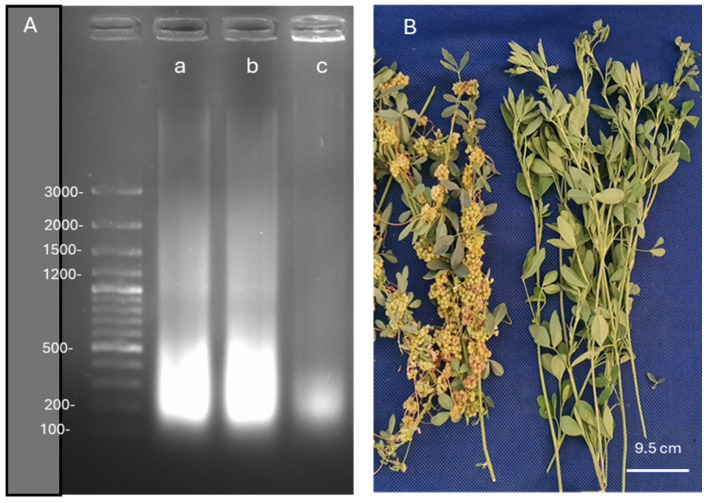
DNA fragmentation images in *Medicago sativa* L. cv. Alsancak, followed by the infection of *Cuscuta* sp. plants (**A**). O’GeneRuler 100 bp DNA Ladder Plus was used to determine DNA fragmentation; 100 µg DNA was loaded for each well, and a size standard is shown on the left. (a) uninfected *Medicago sativa* L. cv. Alsancak (b) *Medicago sativa* L. cv. Alsancak infected with *Cuscuta* sp. (c) *Cuscuta* sp. Infected and control lucerne plants with *Cuscuta* sp. are shown in (**B**).

Lucerne plants followed by the infection of *Cuscuta* sp. showed responses in terms of physiological and biochemical means; most of these symptoms were differentially expressed in leaves and stems. We evaluated whether DNA was damaged or disrupted upon infection by dodders. We found that the damage caused by *Cuscuta* sp. was not severe but evident in infected lucerne plants, as shown in Figure 9A. DNA integrity was disrupted in infected plants when compared to that of the control plant. With a very small amount of DNA fragments of *Cuscuta* sp., we could clearly state that *Cuscuta* sp. preserved its DNA integrity along with the cultivation period of lucerne.

Although not a very big difference was observed between inoculated and control plants in terms of DNA fragmentation, *Cuscuta* sp. succeeded in this infection with very little DNA disruption, as DNA integrity was mostly preserved. It is important to note that lucerne plants carried out their metabolic functions with very little damage; however, this analysis was made at the end of the harvest period, in which the control plants might have shown signs of aging. As observed in Figure 9B, lucerne plants were extensively colonized by dodder plants, and exhibited very weak development, although the intensity of green color as reflected by chlorophyll contents was more or less equal to the non-colonized lucerne plants. However, the aggressiveness of dodder plants was visible through the extensive production of seeds and colonization around the stems of lucerne plants.

## 4. Discussion

*Cuscuta* sp. (dodder) is a holoparasitic plant that extracts water and nutrients directly from its host, severely compromising the host’s growth and development. This parasitism is particularly detrimental to economically important forage crops such as lucerne (*Medicago sativa* L.) and other crop plants and vegetables, leading to substantial yield losses. Attachments of the parasite to the host vascular system via haustoria facilitate the transfer of essential resources, including water, carbohydrates, and minerals [54]. Consistent with previous research, this study confirms that dodder infection markedly affects the anatomical and physiological parameters of lucerne. For example, Förste et al. [55] concluded that the uptake of minerals was selectively regulated. The authors stated that calcium and manganese were depleted in the host. The depletion of Ca ions in host plants led to a reduction in pectin content, which resulted in further penetration of *Cuscuta*. This could partly explain why defense mechanisms are interrupted and suppressed. Similar issues were made with those involved in plant and fungal pathogen interactions. Tian et al. [56] reported that obligate plant pathogens could secrete effector proteins to suppress the plant defense response to make successful colonization. They stated that *Puccinia striiformis* f.sp. *tritici* (*Pst*), one of the most devastating wheat pathogens, secreted effector proteins to suppress plant defense mechanisms. So, it is important to note that here, obligate parasites have the ability to secrete effectors to suppress the host defense. Unlike facultative pathogens, obligate plant pathogens or obligate holoparasitic plants want to control the activities of the host; therefore, they can suppress the host defense depending on the genetic background of the host. Similarly, Xie et al. [57] reported the same mechanism as that of *Meloidogyne incognita* on the suppression of plant defense mechanisms via the *Misp* 12 gene involved in effector biosynthesis. We can enlarge our examples of plant defense suppression. Blaazer et al. [58] reported that several species of herbivorous mites, such as *Tetranychus urticae* and *T. evansi*, maximized their performance by suppressing plant defenses. The more complex issue is underlined by Hettenhausen et al. [59], who stated that metabolites, proteins, and mRNAs are known to be transferred from hosts to *Cuscuta*, and systemic herbivory signals are transmitted from attacked plants to unattacked plants via *Cuscuta* bridges. Some authors suggested that *Cuscuta* spp. induced ethylene in susceptible crop cultivars, including vegetables such as tomato and tobacco plants [60]. This pathway may have been involved in the suppression of the defense responses of *M. sativa*. However, more pathways are needed other than hormone signaling to reveal the mechanisms underlying defense suppression.

Saric-Krsmanovic et al. [18] reported significant anatomical alterations and physiological impairments in infected plants. The findings are further supported by field studies that show dodder infestation weakens host plants, resulting in decreased growth and reproductive capacity [61]. Notably, dodder parasitism has been associated with reduced dry matter accumulation, thereby negatively impacting yield and biomass production, as shown in Figure 6, Figure 7 and Figure 8. We observed that the infestation and seed production of *Cuscuta* sp. exhibited low defense responses of lucerne plants.

At the molecular level, transcriptomic analyses revealed that dodder infection downregulated photosynthesis-related gene expression and disrupted protein synthesis in the host [62]. This downregulation is reflected physiologically by a significant decrease in photosynthetic capacity in lucerne during infection [10,18]. The parasite also induced activation of host defense-related genes and signaling pathways, which further suppressed photosynthetic activity [63]. Consequently, the reduction in photosynthesis adversely affects the protein synthesis, which correlates with decreased chlorophyll content—including total chlorophyll, chlorophyll *a* and *b*—in infected tissues [64,65]. Energy spent on defense purposes costs the quality parameters, as shown in the work of Baran et al. [52]. Moreover, dodder infection significantly disrupts photosystem II activity and other chlorophyll fluorescence parameters, indicating compromised light-harvesting efficiency and photosynthetic electron transport [66]. This reduction in photosystem II function is likely linked to diminished chlorophyll levels and disruption of the light-harvesting chlorophyll–protein complex II [64,67]. The suppression of photosynthetic protein gene expression coupled with the activation of defense responses highlights a complex host–parasite interaction, where energy production is sacrificed to mount an effective defense.

Lucerne serves as a major source of animal fodder; the observed decline in protein synthesis and photosynthetic efficiency has important implications for both crop yield and nutritional quality. Protein reduction in parasitized leaves adversely affects forage quality, directly impacting livestock nutrition and productivity. Farah and Al-Abdulsalam [68] reported yield losses exceeding 50% in heavily infected fields, underscoring the significant economic threat posed by dodder. Overall, these findings highlight the multifaceted impact of *Cuscuta* sp. on lucerne, involving anatomical damage, physiological dysfunction, and molecular disruptions that collectively reduce host vitality and productivity. Future research should focus on elucidating the molecular mechanisms underpinning host resistance and parasite virulence. Such insights could facilitate the development of resistant lucerne cultivars or innovative management strategies to mitigate the detrimental effects of dodder parasitism.

Phenolic compounds are secondary metabolites that play a crucial role in plant defense mechanisms against various biotic stress factors, including parasitic infections. These compounds function as direct antimicrobial agents against pathogens, participate in signal transduction processes, and contribute to the reinforcement of cell walls through lignin biosynthesis, thereby forming a physical barrier [69]. However, several studies have reported that *Cuscuta* sp. infections suppress phenolic metabolism or reduce the levels of these compounds in host plants. For instance, Runyon et al. demonstrated that phenolic accumulation was quite low, followed by the suppression of the pheylpropanoid pathway in tomato plants infected by dodder pentagona [70]. Similarly, Albert et al. [8] reported that dodder species can actively suppress host defense responses, particularly phenolic compound production, to ensure successful parasitism. In the present study, a significant reduction in phenol content was observed in both leaf and stem tissues of infected lucerne plants, suggesting that dodder remarkably weakened the host defense systems, as shown in Figure 3. This suppression may be mediated through the disruption of crosstalk between phytohormones, especially ethylene, SA, and JA, which play a key role in the biosynthesis of phenolic compounds [71]. Moreover, parasitic plants such as dodder have been reported to alter phenolic accumulation and inhibitory activity in host tissues, thereby compromising the host’s defense capacity [72].

Parasitic plant infections disrupt the oxidative balance in host plants by inducing metabolic stress and promoting the accumulation of reactive oxygen species (ROS). Under such biotic stress conditions, plants activate tissue-specific antioxidant defense mechanisms to maintain cellular homeostasis. The findings of this study demonstrate that enzymatic responses to dodder infection vary depending on the tissue type, reflecting differential regulation of stress signaling across plant organs. A notable increase in CAT activity observed specifically in stem tissues suggests a critical role for this enzyme in ROS detoxification. Previous studies have reported that CAT activity increases during certain developmental stages in plant–pathogen interactions to transiently regulate intracellular H_2_O_2_ levels [73]. The elevation of CAT activity in stems of dodder-infected plants may represent a localized defense response aimed at preserving tissue integrity, especially at the site of haustorial invasion. In our study, we observed the defense responses more commonly around the infection sites. Patterns of antioxidant enzymes, Figure 4 and Figure 5, exhibited that the defense responses were around the invaded sites, especially in stem sections, compared to those of leaves. Fareed et al. [26] reported that in the non-parasitized sites on the tomato stems, SOD and CAT activities remained relatively low and consistent when compared to those of *Cuscuta chinensis*-infected sites. The authors clearly stated that there was a significant increase in SOD activity in the parasitized sites. Conversely, at the non-parasitized sites, there was a comparatively lower CAT activity. Fareed et al. [26] also reported that there was a comparatively lower but still elevated malondialdehyde (MDA) content at the non-parasitized sites. They suggested that even areas not directly impacted by parasitism still had oxidative stress, but the infected sites had more stress metabolites. Similarly, we found that a significant increase in POX activity, again limited to stem tissues, highlights another key component of the host defense system. POX enzymes are involved in lignin biosynthesis and cell wall strengthening, processes that contribute to the formation of structural barriers against invading pathogens [74]. The increased POX activity observed in infected regions has been linked to activation of the phenylpropanoid pathway and suggests a defense-induced remodeling of cell walls [75]. However, in leaves of lucerne plants, increases in antioxidant enzymes ceased, indicating that defense responses were not carried out further. These tissue-specific increases in CAT and POX activities imply that the host plant develops a localized defense strategy in response to dodder infection. Given that the stem is the primary site of haustorial penetration and resource extraction, the enhanced activation of antioxidant enzymes in this region appears to be a biologically adaptive response. In contrast, the absence of significant enzymatic changes in the leaf tissue may indicate that the defense response is concentrated at the infection site and that systemic responses are either limited or delayed.

Jasmonic acid (JA) and its derivatives, along with salicylic acid (SA), are key regulators of plant defense responses against diseases and environmental stress factors [29]. The effects of changes in JA levels on plant defense responses are quite complex and multifaceted [76]. In particular, increases in JA levels enhance the expression of defense-related genes, thereby promoting the synthesis of defense metabolites in plants [77,78]. Plants regulate their defense responses against environmental stress and pathogen attacks not only through JA but also via other hormonal signaling pathways, such as SA, ethylene, and abscisic acid. Therefore, even if a significant increase in JA levels is not observed, effective defense responses can still be activated through these alternative pathways. Moreover, JA responses are typically rapid and transient; JA levels may rise shortly after stress exposure and quickly return to baseline [79]. The synthesis and degradation of JA are tightly controlled by negative feedback mechanisms, preventing unnecessary accumulation. As a result, stable JA levels may be detected at the time of measurement. This dynamic regulation ensures efficient use of the plant’s energy resources and maintains hormonal balance. On the other hand, SA is a phenolic phytohormone involved in key physiological processes such as photosynthesis, transpiration, and the uptake and transport of ions [80,81]. It also plays a crucial regulatory role in shaping leaf anatomy and maintaining the structural organization of chloroplasts [82,83]. The synthesis, accumulation, and metabolism of SA can rapidly fluctuate depending on environmental conditions [84]. Derivatized forms of SA produced during these processes may not always be detectable, which can result in the absence of a noticeable change in total SA levels [85]. Moreover, plants can activate their defense responses not only through SA but also through other phytohormones, defense proteins, low concentrations of ROS, and phytoalexins, all of which can take significant roles [86,87]. In certain plant–pathogen interactions, SA signaling may be suppressed or activated with a delay. Our results indicate that dodder infection elicited tissue-specific oxidative stress responses in the host plant. The observed enzymatic changes might have taken significant roles in the regulation of ROS levels and structural changes in infected tissues to counteract parasitic invasion, as shown in Figure 5. However, our findings showed that there was no increase or elevated levels in JA or SA, indicating that defense responses were not carried out along with the cultivation season. Shi et al. [88] stated that SA was involved in the resistance of tea plants to anthracnose infection caused by *Colletotrichum* spp. They reported that the total amount of SA was approximately twice as high in anthracnose-infected tea leaves compared to healthy leaves. Susceptible tea cultivars were not able to exhibit high SA content. Therefore, the determination of JA and SA contents in infected and non-infected plants could be a good criterion to elucidate the defense responses and screening tests for the resistance of different cultivars to a particular type of stress.

Parasitic infections induced localized anatomical changes in the host and partially suppressed the defense mechanisms. Prominent callose formation was observed at the sites where *Cuscuta* sp. attached to the host plant. This suggests that the plant attempts to limit parasite progression by creating a physical barrier through localized cell proliferation at the infection site. The literature frequently emphasizes that callose tissue develops as a mechanical and chemical defense response against parasitic plants [89,90]. The intense callose formation in stem tissues particularly highlights the critical role of localized defense, given that dodder haustoria obtain nutrients through this region. In conclusion, callose development represents an effective tissue-level defense strategy employed by the host plant against dodder infection.

When the DNA fragmentation assay was performed, we observed that *Cuscuta*-infected *M. sativa* accumulated slightly more low-molecular-weight DNA molecules when compared to control groups. Although *Cuscuta* sp. did not cause significant DNA damage, low levels of DNA damage could disrupt the whole metabolism. The progress of DNA damage was evaluated through a timescale experiment to determine if recovery takes place. Disturbance in DNA molecules could be very costly for the host plant. For example, Turan et al. [91] reported that DNA fragmentation in spermatozoa affected the fertility in men. Similar issues were made by Kaya et al. [92] who stated that high-dose vanadium resulted in DNA fragmentation in *Allium* plants and significantly disturbed the metabolism. Again, Plitta-Michalak et al. [93] showed that the loss of DNA integrity disrupted the whole metabolism and either delayed or prevented the recovery of *Acer pseudoplatanus* L. Under abiotic stress, the progress of *Cuscuta* could lead to more devastating consequences. For example, Demirbas et al. [94] reported that *A. thaliana* seedlings exposed to salinity were more sensitive to *Phelipanche ramosa* when compared to the uninoculated plants, indicating that there was a positive correlation between salt sensitivity and the ability of *P. ramosa* to infect *A. thaliana* plants. The defense barrier was further suppressed. Therefore, resistant plants could not defend themselves when exposed to multiple stressors. Analysis of host defense response suppression under these circumstances may reveal what genes and metabolites are responsible.

## 5. Conclusions

Understanding the biochemical and molecular pathways involved not only in the pathogenicity of holoparasites but also in desiccation, temperature stress, UV radiation, and salinity is essential for controlling parasitic weeds. The majority of the research has been conducted on parasitic plants and their hosts; however, stress-tolerant parasitic plants may threaten agricultural production, followed by climate changes that could modify irrigation regimes and cause drought, salinity, and other environmental stresses. Under these circumstances, tolerance or resistance levels of host plants break, and defense suppression could be easier for the stress-tolerant holoparasitic plants. To better understand the defense suppression of host plants followed by holoparasitic infection, this study formed a strong database to evaluate the behavior of holoparasites under stress conditions. Our findings demonstrated that dodder parasitism in lucerne triggered significant declines in chlorophyll content, photosynthetic efficiency, and protein synthesis, alongside notable structural modifications in host tissues. The observed suppression of phenolic compounds, which are key components of plant defense, suggests that dodders strategically impair the hosts’ protective metabolic pathways—particularly those associated with the phenylpropanoid biosynthetic route and phytohormonal crosstalk involving SA and JA. Concurrently, a tissue-specific oxidative stress response was recorded with elevated CAT and POX activities in stem tissues and decreased or unchanged activities in leaves, indicating a localized antioxidant defense strategy. If we could reveal the hormone mechanisms in detail, we could then inhibit hormones that promote haustorium formation to suppress the invasions of parasitic plants.

Histological analysis further revealed prominent callose formation at the penetration sites. This localized cell proliferation likely serves as a physical barrier against parasitic invasion and represents a structural manifestation of host defense. The intensity of callose development in stem tissues underscores the biological importance of localized defense responses in regions directly targeted by the parasite.

As DNA fragmentation was examined, the infected plants showed signs of DNA damage. The observation regarding DNA damage was obtained at the very end of the trial, but some recovery might have occurred. In our coming studies, we will observe DNA integrity at earlier times with more frequent time intervals to elucidate the threshold level of tolerance of the plant against *Cuscuta* infection.

Collectively, the data suggest that dodder infection elicits a complex interplay of localized and systemic responses in lucerne involving both the suppression and activation of defense pathways. Understanding these intricate host–parasite interactions is critical for developing resistant lucerne cultivars and designing effective management strategies to mitigate the impact of dodder infestation in agricultural systems.

If defense-related genes could show an increase or stability, we could follow that pathway to generate or breed multiple stress-tolerant plants, including those resistant to holoparasitic plants; if not, we could come back to square one and start generating multiple stress-tolerant crop plants after revealing all pathways. If tolerance- or resistance-related defense genes and metabolites are downregulated, the only way to generate more tolerant or resistant crop plants is to reveal whole plant metabolism. Therefore, breeding tasks would be more difficult as the stress becomes more complex.

## Data Availability

The original contributions presented in this study are included in the article. Further inquiries can be directed to the corresponding author.

## Abbreviations

The following abbreviations are used in this manuscript:

CAT	Catalase
POX	Peroxidase
JA	Jasmonic acid
SA	Salicylic acid
Fwt	Fresh
Dwt	Dry
HGT	Horizontal gene transfer
